# Residents’ Location-Based Fear of Theft and Their Impact Factors in Guangzhou, China

**DOI:** 10.3390/ijerph20010638

**Published:** 2022-12-30

**Authors:** Guangwen Song, Jiaqi Li, Chunxia Zhang, Jie Gu

**Affiliations:** 1 Center of GeoInformatics for Public Security, School of Geography and Remote Sensing, Guangzhou University, Guangzhou 510006, China; 2 School of Tourism and Business, Guangzhou Panyu Polytechnic, Guangzhou 511483, China; 3 College of Architecture & Urban Planning, Hunan City University, Yiyang 413000, China

**Keywords:** fear of theft, location-based, correlation factors, Guangzhou

## Abstract

While the fear of theft is common and is known to lead to lower satisfaction with life and subjective well-being, current literature regards the fear of theft as a stable psychological state and ignores discrepancies based on location and their influencing factors. To fill these gaps, we selected 74 typical communities and collected 1568 questionnaires throughout Guangzhou. The results show that: (1) the respondents demonstrated significant location-based differences in their fear of theft. Locations including a coach station, a railway station, a bus station, a subway station and a wholesale market had the highest associated levels of fear, whereas locations dedicated to leisure activities, especially those in high-end places, had a lower level of respondents’ fear of theft. (2) Vulnerability model, victimization model, community security and built environment can be applied to the analysis of fear of theft around different places, but interpretations of fear do vary widely from place to place.

## 1. Introduction

The fear of crime is a prevalent issue in modern society and is closely related to residents’ satisfaction with daily life [1,2,3]. Since the mid-1960s in the west, the sociological study of this fear has emerged as an important research area and has since developed into an interdisciplinary research field [4].

Literature on the fear of crime is concentrated in western nations, specifically in the United States and in other English-speaking nations. With increased interest in this field of research, some scholars from non-western countries have begun to study the fear of crime from cross-cultural and intercultural perspectives. As the scope and depth of this research grows, new research from more diverse cultural backgrounds has emerged. For instance, some Turkish studies have found that the integrated model of the fear of crime that was developed by western scholars functioned differently in a Turkish local context, based on gender and residential locale [5]. Likewise, literature from South Korea found that district-level crime rates negatively correlated with all types of neighborhood associations, but the variation between violent crime and property crime was not obvious [6]. Much Mexican research explores the impact of criminal violence in Mexico on the population’s mental health and concludes that the activities of drug trafficking organizations (DTOs) have led to a sharp increase in related violence [7]. Relevant literature from China has studied the influence of factors such as hukou (household registration) and Guanxi (individual social networks) on fear of crime [8,9]. According to the conclusions of existing cross-cultural studies, the influencing factors of fear of crime vary to some extent based on different cultural backgrounds. Compared with the voluminous research from western countries, the quantity of non-western studies is still very limited. However, it is useful to review the findings on the fear of crime in diverse contexts as some elements will vary across nations.

Location is a significant factor in predicting crime; similarly, the fear of crime is location specific. Routine activity theory posits that a motivated perpetrator, potential victim and absent supervisor are the three necessary conditions for crime to occur [10] and that crime is not evenly distributed in space [11,12]. Furthermore, according to the rational choice theory, a crime is the result of a criminal’s rational and comprehensive judgment of risks, benefits and costs. In short, criminals choose the location of their crime based on an evaluation of the risks and benefits of the surrounding social environment [13]. Previous studies on the fear of crime have interpreted the fear of crime as a stable psychological state without spatial variation. However, ignoring the spatial differences involved in the fear of crime is problematic because location variations such as activity type, social class and population transience are assumed to have an impact on residents’ fear of crime [14].

Moreover, most existing literature has had significant difficulties in identifying types of crime when attempting to assess the fear of crime [15], and they often use vague questions to measure fear, which do not involve specific crime types (such as theft, fraud, burglary, etc.) or locations [16,17,18]. Furthermore, in literature that does address the type of crime, the majority of them focus on the fear of violent crime [19]. However, the fear of crime is not a universal and featureless fear; crime type and location must be addressed, or they may inadvertently measure other contemporaneous issues or phenomena. With this in mind, we look to theft as one of the most common types of crime that affect people’s daily lives and have gained much attention from the literature [20]. As such, this paper studies the specific fear of theft.

To sum up, it is necessary to analyze how location factors affect individual fear of crime and its influencing factors. This paper questions whether there are spatial differences in the fear of thefts and further examines whether there are significant variations in influencing factors for fear of theft around different locations.

## 2. Literature Review

### 2.1. Definition and Measurement of Fear of Crime

Due to the variety of research perspectives and academic fields that work with the fear of crime, there are a number of extant definitions. For example, Conklin defines fear of crime as the perception that an individual is being victimized in a community, whereas Garofalo defines fear as an emotional reaction characterized by a sense of danger and anxiety [21,22]. Garofalo’s definition is restricted to feelings of danger and anxiety produced by the specific threat of physical harm. Similar to Garofalo, Ferraro argues that the fear of crime is emotional anxiety stimulated by fear of death or of signs associated with a criminal activity [23].

The opposite of the fear of crime, a sense of security is a similarly important concept in psychological research. In western criminology research, a sense of security is regarded as the binary opposite of fear of crime. Psychologist Robert Maslow, who developed the Security-Insecurity Questionnaire, defines psychological security as a feeling of confidence, security and freedom from fear and anxiety when addressing one’s present and future needs [24].

All measurements and subsequent analysis of the fear of crime are based on questionnaires and generated by different researchers. This also leads to differences in how researchers understand the fear of crime. For instance, in 1971, Furstenberg measured the fear of crime in two ways [25]. First, respondents were asked to select the single most serious domestic problem, from a list of ten, that they would like to see the government do something about. Second, respondents rated the possible risk of their being victimized for eight different offenses. In 1975, Lebowitz took a different approach and measured the fear of crime using a single question (is there any area right around here—that is, within a mile—where you would be afraid to walk alone at night?) [26]. In 1983, Baker et al. took up the notion of security at night by measuring the fear of crime with two questions [27]. First, how safe you feel walking alone at night in your neighborhood. Second, respondents were asked to think of the worst area within a mile of their homes and to rate how safe they felt walking alone at night in that area. However, there is little consistency between researchers and their methods; Thomas and Hyman measured fear of crime with nine questions, and other researchers [28] focus on the fear of crime as an event experienced in a particular situation [29]. More recently, Gabriel and Greve (2003) argued that the fear of crime includes three conditions: the individual’s cognitive perception of victimization (cognitive dimension), a corresponding emotional experience (affective dimension) and an appropriate motivation and behavioral tendency [30].

Therefore, the definition of fear of crime can be summarized into three dimensions, namely the emotional dimension, the cognitive dimension and the behavioral dimension, which can be investigated through questionnaires. Researchers can use a series of questions or only one single question to measure respondents’ fear of crimes.

### 2.2. Correlates of Fear of Crime

In existing literature on the fear of crime, there are a number of important corollaries that arise in proximity to that fear. These influencing factors have been extensively modeled and can be summarized as follows: the victimization model, the vulnerability model, the disorder model, the collective effectiveness model and the community policing model [31].

The victimization model posits that previous victimization and perceived severity of victimization make individuals more sensitive to crime, which generates higher levels of fear [32]. Two hypotheses undergird victimization model: first, those who are prior victims of property or violent crimes will display higher levels of perceived risk than nonvictims. Second, victims who perceive their victimization as a more severe event will display higher levels of perceived risk than those who consider an instance of prior victimization to be less severe. Empirical research shows that both the initial severity of victimization and the subjective perception of that victimization are impact factors for predicting later fear of crime [33]. Furthermore, studies show that the experience of crime does not have to be direct: indirect infringement such as hearing about the victimization of other local victims can also enhance residents’ fear of crime [34,35]. However, there is only a weak correlation between indirect prior victimization experience and residents’ fear of crime [36].

The second model is the vulnerability model, which holds that fear of crime is closely related to individual vulnerability. For example, although studies show that women are less vulnerable to violent crime than men, they have a higher perception of crime risk because they are more physically vulnerable than men [37]. In terms of age, vulnerability increases with age, and the fear of crime among the elderly is often higher than that among the young [38]. Similarly, in terms of the economy, low-income residents are more economically vulnerable than high-income residents, so they have a higher rate of fearing crime [39]. However, while interesting, the conclusions of the vulnerability model are not always valid or accurate. Some recent studies found that vulnerability is not significantly associated with the fear of crime, and even reached a contrary conclusion with respect to age [40]. Both the vulnerability model and the victimization model work from the perspective of the individual but fail to consider context outside of prior victimization or demographic information.

Contrastingly, the disorder model and the collective effectiveness model introduce analyzing the fear of crime from the perspective of an individual’s residential environment [41]. In 1961, Jacobs proposed the ‘eyes on the street theory’ in her book, The Death and Life of Great American Cities, and argued that high-density buildings, mixed land use and a compact layout within the built environment created conditions that enhanced the mutual protection of people and that helped to reduce the fear of crime [42]. Jeffery (1971) proposed the ‘Crime Prevention Through Environmental Design’ (CPTED) theory, which posited that there exists a causal link between environmental stimulus, crime, and fear of crime, and that environmental design can inhibit the occurrence of criminal events to some extent [43]. On the basis of the CPTED theory, Wilson and Kelling proposed the Broken Windows theory, which used broken windows in a community to emphasize the intrinsic correlation between community environmental disorder, criminal incidences and the fear of crime [44]. The disorder model has since been widely used to explain the fear of crime in some communities [45]. In contrast to the disorder model, collective efficacy posits that a neighborhood with a high level of collective efficacy may reduce social disorganization and reduce resident’s fear of crime [46]. Nevertheless, the disorder model and the collective effectiveness model, which highlight the importance of social environment, tightly relate with individuals’ fear of crimes within their residential neighborhood but fail to explain their fear of crime in public places out of their living places. Instead, the physical environment in neighborhood can increase individuals’ knowledge about varied activities in different places [47], therefore impacting their safety perceptions around different places.

Therefore, in this study, we will take indicators of vulnerability, victimization experience, community security conditions as well as physical environment into consideration and innovatively explore their impacts on the fear of theft around specific places.

## 3. Data and Methodology

### 3.1. Data

Research for this study was conducted in Guangzhou, the third largest city in China, which is located in the south of the country. Guangzhou is composed of 11 districts, namely Liwan, Yuexiu, Haizhu, Tianhe, Baiyun, Huangpu, Panyu, Huadu, Nansha, Conghua and Zengcheng. According to the Statistical Yearbook of Guangzhou, in 2020, the administrative area of Guangzhou covered 7434.40 square kilometers, the permanent population at year-end totaled 18.74 million, the registered permanent residents at year-end totaled 9.85 million and Gross Domestic Product generated over the course of the fiscal year came to USD 373.54 billion. The geographical scope of the questionnaire survey included the entirety of Guangzhou city except for the Conghua district and the Zengcheng district. These two districts were excluded because they are administratively under the jurisdiction of Guangzhou, but they are geographically remote and are far away from the central city.

**Figure 1:**
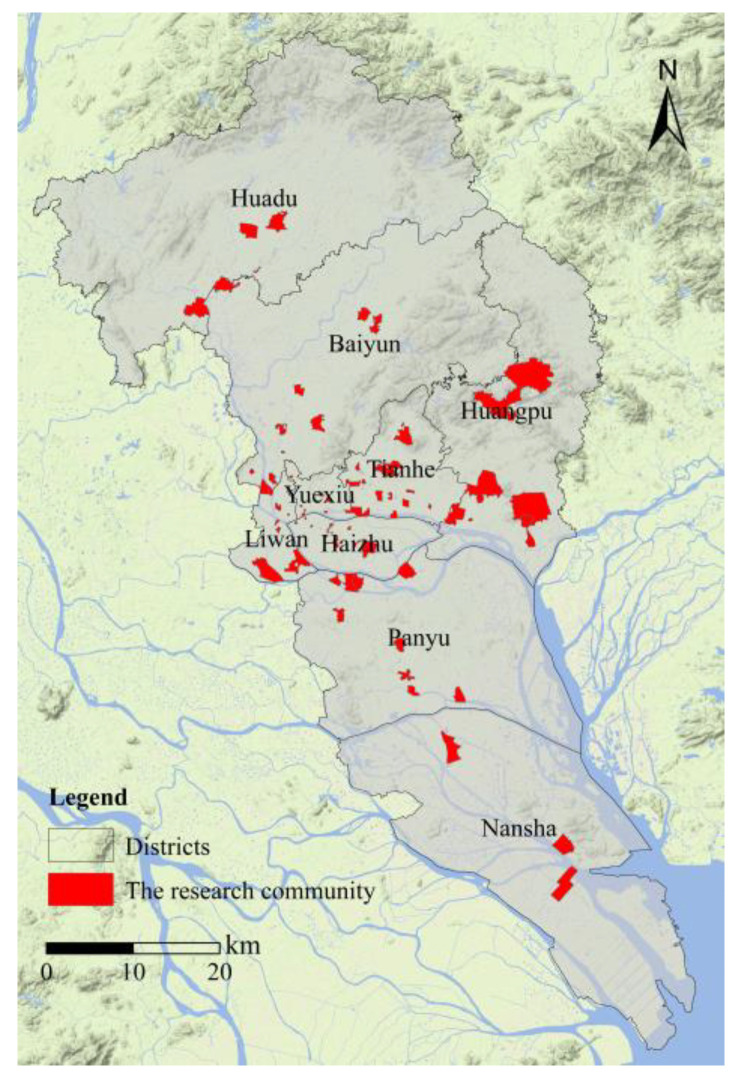
Communities that participated in the questionnaire across Guangzhou.

We obtained our data through a Questionnaire Survey of Guangzhou Community Environment and Residents’ Safety Perception Project. The survey was conducted from January to April 2016 (excluding the Spring Festival). The respondents were all permanent residents over the age of 18 (excluding school students), and the content of the questionnaire was broken down into following parts: filtering topics (interviewees who do not meet the research requirements shall terminate the interview), basic personal information, basic housing information, basic community information, community safety and satisfaction survey, crime reporting rate and safety perception. There are a total of 1501 communities located within the districts of Guangzhou (excluding Zengcheng and Conghua) (Figure 1). A total of 74 communities were selected to participate in the questionnaire survey.

This study draws its principal components from the 6th national population census, and clustering was conducted to identify the types and characteristics of communities. After several rounds of screening and comparison, 40 indexes in 10 categories were selected for orthogonal rotation factor analysis and cluster analysis. The clustering results include nine types of communities: middle-class communities, college communities, non-aging rural communities, old city aging communities, aging rural communities, migrant communities, low-rent housing communities, affordable housing communities and high-end residential communities. Based on the types of community, we firstly allocate the sample size to each type of community according to the proportion of the population of such communities to the total population and then considered their spatial distribution, and we selected typical communities for field investigation and carrying out this questionnaire survey.

The questionnaire was distributed randomly, and the interval between each of the interviewees was required to be more than five households. At the end of the first round of the investigation, the validity of the questionnaire was tested and a supplementary survey was conducted to eliminate any unqualified questionnaires. After this process, 1568 out of 1700 questionnaires were valid and used in this study.

### 3.2. Measures

#### 3.2.1. Dependent Variable

The dependent variable is the fear of theft, which was derived directly from the questionnaire. Existing literature adopts two methods to measure the fear of crime. The first is to generate multiple sub-items and use the principal component method to extract the principal factor to measure [48]. The other is to use a single item as the measurement, that is, the comprehensive score of the crime fear directly investigated [49]. This study deployed the second method to measure the fear of theft. Respondents were asked the following question: “Are you afraid of being a victim of theft around the following locations?”. The responses ranged from “Not afraid at all” to “Very afraid” on a scale from 1 to 5. In total, we investigated their fear of theft around 16 kinds of places: (1) parks, greenways and other recreational areas; (2) sports venues and surrounding areas; (3) internet cafes; (4) bars; (5) KTV and cinemas; (6) high consumption restaurants; (7) medium consumption restaurants; (8) low consumption restaurants; (9) urban villages; (10) a bank and its surroundings; (11) everyday shopping places; (12) commercial streets/shopping centers; (13) food markets; (14) bus stations and subway stations; (15) wholesale markets; (16) coach stations and railway stations.

Table 1 shows that, on average, the places with the lowest fear of theft were high consumption restaurants (average score: 2.543), sports venues and medium consumption restaurants (average score: 2.604) and low consumption restaurants (average score: 2.710). The places with the highest fear of theft were coach stations and railway stations (average score: 3.940), urban villages (average score: 3.572) and bus stations and subway stations (average score: 3.477), all of which have a highly transient population and high levels of anonymity.

We used the factor analysis method to extract the principal components as the dependent variables. The *p* value of Bartlett sphericity test probability is less than 0.001, indicating that there are significant differences between the prior relational number matrix and the identity matrix. The KMO (Kaiser- Meyer- Olkin- Measure of Sampling Adequacy) value is 0.910, and according to the KMO metric standard, the original variables are suitable for factor analysis. Interpretation of the total variance of the factor analysis shows that the characteristic root of the first factor is 6.959, which explains 43.5% of the total variance of the 16 original variables. The characteristic root of the second primer is 1.784, which explains 11.2% of the total variance of the 16 original variables and brings the total cumulative variance to 54.65%. The characteristic root of the third introduction is 1.191, which explains 7.4% of the total variance of 16 original variables, bringing the cumulative variance contribution is 62.1%. Finally, three principal factors were extracted.

Table 1 outlines that the variables in leisure places such as parks and greenways (*Y*_1_–*Y*_7_, *Y*_12_) have a high load on the first factor. This shows that factor 1 encapsulates the interviewees’ fear of theft around leisure and entertainment spaces and middle- and high-end consumption spaces. Therefore, factor 1 can be called “fear of theft around leisure activity places”. The second factor has a higher load on variables such low consumption restaurants and urban villages (*Y*_6_–*Y*_13_, *Y*_16_). Therefore, factor 2 encapsulates the interviewees’ fear of theft during daily activities in places of middle- and low-grade prices of consumption, and it can be called “fear of theft around mid- to low-end places”. Variables such as the bus station, subway station, wholesale market and train station have higher loads on the third factor (*Y*_11_, *Y*_14_–*Y*_16_). Therefore, factor 3 encapsulates the interviewees’ fear of theft in transportation spaces and wholesale markets, and it can be called “fear of theft in high mobility places”.

#### 3.2.2. Covariates

In this study, we divided the influencing factors of fear of theft into vulnerability, victimization experience, community security and built environment. Vulnerability variables include physical vulnerability variables and social vulnerability variables. Gender (dummy variable: male = 1; female = 2) and age (continuous variable) were included as indicators of physical vulnerability. Social vulnerability variables include marital status, household income and hukou. Marital status (dummy variable: married = 1; unmarried, divorced or widowed = 2) provided a measure of social relations. Household income (ordinal variable: low income to high income coded from 1 to 6) provided a measure of the wealth of each respondent. Hukou, the status of household registration, was designed as a dummy variable in our survey, 1 for local hukou, and 2 for non-local hukou. Non-local hukou includes rural and urban migrants to Guangzhou who did not have local hukou status when the survey was conducted. People with local hukou often have a higher income and better urban social welfare.

The second influencing factor was prior experience with victimization. Victimization experience was indicated by whether the respondent had been a victim of crimes in the last three years preceding the survey. There were 5 variables on crime victimization experience. The question was “Have you experienced any of the following crimes in the past three years (violence in community, theft in community, burglary, theft in bus or subway and theft in public places)?”. The possible answers were: 1 for zero times; 2 for 1–3 times; 3 for 4–6 times; 4 for 7–9 times; 5 for more than 10 times.

The third type influencing factor was the community security environment. In this study, the quality of a community’s security environment was measured based on whether the community had a crime occur within the three years preceding the survey. The question was “Have any of the following crimes been committed in the community in the past 3 years?”. The variable type was a dummy and crime types were divided into five categories, each with the option of no or not heard of it = 1, or yes = 2.

The fourth influencing factor was the built environment. The characteristics of built environment were measured by POI (Point of Interest), meaning the density of 12 types of public service facilities or commercial facilities, including bars, restaurants, hotels, night clubs, cinemas, entertainment facilities, scenic spots, stores, arts and culture venues, stadiums, banks and security facilities. This study crawled the points of interest in the 74 participating communities from a map website, then divided the number of POI in each category by the area of the community to obtain the density of points of interest in units per square kilometer. These POIs play important roles in the spatial patterns of thefts [47] and therefore may impact respondents’ location-based fear of theft.

**Figure 2:**
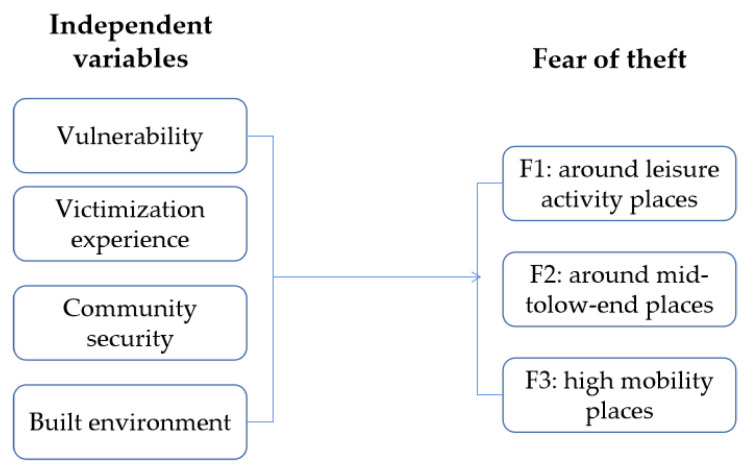
The conceptual framework of the relationship among independent variables and fear of theft around different location.

The conceptual framework of this study can be concluded in Figure 2.

### 3.3. Analytic Strategy

A multiple linear regression model was adopted to determine the correlation between independent variables and the fear of crime. The empirical analysis model was constructed as follows:

(1) $$Y_{i}=a+\overset{k}{\underset{j}{\sum }}\beta_{ij}X_{ij}+\varepsilon_{i}$$

In Formula (1), *Y_i_* represents the principal component factor of the fear of theft Respondent *i*; *X_j_* is the independent variable. *β_ij_* is the regression coefficient of the independent variable *X_j_* of Respondent *i*, indicating that with every change in a dependent variable indexed by one unit, the fear of theft will change by *β*. The a is a constant term; the *ε_i_* represents the error term, which includes those factors that are not considered in the model but that may affect a respondents’ fear of theft. The variance inflation factors (VIF) of the independent variables are less than 5, indicating that there is no serious collinearity problem among the variables. All analyses were performed using SPSS 22.0 software.

## 4. Results

### 4.1. Descriptive Statistics of Independent Variables

The dependent variables and correlates of the fear of theft are displayed in Table 1 and Table 2. In terms of gender, male respondents accounted for 53.3% and female respondents accounted for 46.7%. The respondents were 19 to 89 years old, and the average age was 38.030 years (SD = 14.016).

### 4.2. Model Results

Altogether, there are three models of linear regression (Table 3). The dependent variables in Model 1, Model 2 and Model 3 correspond to *F*1, *F*2 and *F*3, respectively. The *p* values of the *F*-test statistic in the three models are 0.000, indicating that the models are reasonable and there is a linear relationship between the independent variables and the corresponding dependent variables of these three models.

(1) The impacts of vulnerability variables

Among vulnerability variables, three variables (*X*_1_, *X*_3_ and *X*_4_) are significant in Model 1, only one variable (*X*_2_) is significant in Model 3, and no variables are significant in Model 2.

In Model 1, there is a strong positive correlation between gender and *F*1. These results show that female residents have a significantly higher fear of theft around leisure activity places than their male counterparts. In Model 2 and Model 3, such linear relationships between gender and dependent variables are not significant. Contrastingly, age had a significant negative impact on *F*3, the fear of theft around mid- to low-end places. As age increased, fear of theft presented a downward trend. Nevertheless, the impact of age is not significant in *F*1 and *F*2. In terms of household income, it significantly increased the fear of theft around activity places but showed no significance in Model 2 and Model 3.

In Model 1, the linear relationship between marital status, income and dependent variables (*F*1) is significant, and there is a positive correlation between household income and *F*1 while such correlation is negative for marital status. In Model 2 and Model 3, the linear relationship between marital status as well as household income and dependent variables showed no significance. In addition, hukou registration had no significance across all three models.

(2) The impact of victimization experience

Among the variables of prior experience with victimization, three variables (*X*_6_, *X*_8_ and *X*_9_) are significant in Model 1, only one variable (*X*_8_) is significant in Model 2, and no variables are significant in Model 3. Among the three significance variables of Model 1, the regression coefficients show that *X*_6_ and *X*_8_ are negatively correlated with *F*1. The regression coefficient of *X*_9_ is greater than 0, indicating that this variable has a positive correlation with *F*1. In Model 2, the regression coefficient of *X*_8_ is greater than 0, indicating a positive correlation between this variable and *F*2.

(3) The impact of community security

The respondents’ knowledge of criminal incidents in the community was used to measure community security. In Model 1, the relationship between *X*_12_ (positive) and *X*_15_ (negative) and *F*1 is significant. In Model 2, there is a positive significant relationship between rape or murder (*X*_15_) and *F*2. Lastly, in Model 3, the positive relationship between burglary is significant, indicating that residents who have heard about burglary in the community have a higher rate of fear of theft in high mobility places.

(4) The impact of built environment

In Model 1, there is a positive significant relationship between entertainment facilities (*X*_21_) and *F*1. In Model 2, there are five indicators with significant relationships with *F*2, among which the regression coefficients of *X*_17_, *X*_18_, *X*_20_ and *X*_26_ are less than 0, indicating that these 4 indicators are negatively correlated with *F*2. The regression coefficient of *X*_21_ is greater than 0, indicating that these indexes are positively correlated with *F*2. In Model 3, *X*_17_, *X*_20_ and *X*_25_ have significant negative impacts on residents’ fear of theft in high mobility locations. Contrastingly, the presence of *X*_21_ and *X*_24_ significantly increased the residents’ level of fear of theft.

## 5. Discussion

The goal of this study was, in part, to contribute to the analytical framework of fear of crime in the non-Western context from the perspective of locations. Overall, the respondents’ fear of theft demonstrably varied based on location and correlation factors highlighted significant differences. 

The respondents showed significant location-based differences in their fear of theft. High mobility locations, including coach stations, railway stations, bus stations, subway stations and wholesale markets had the highest level of fear of theft, associated with them, which seemed due to the largely transient population with high levels of anonymity and mobility. Conversely, people feel less worried about theft around leisure activity places, especially high-end places, which had higher levels of guardianship and put people at ease. Locations in the middle to low end had relatively high fear levels associated with theft. The results of factor analysis verify these conclusions and indicate that location-based fear of theft can be summarized into three categories: *F*1 standing for the fear of theft in recreational and leisure facilities and high-consumption places; *F*2 for daily activity places and middle and low consumption places; *F*3 for mainly public transport places and commodity wholesale places. *F*1, *F*2 and *F*3 can be, respectively, named: fear of theft around leisure activity places, fear of theft around mid- to low-end places and fear of theft around high mobility places.

There are various location-based impact factors that influence the fear of theft. Prior experience of victimization, including violence and burglary, as well as rape or murder happening in community would decrease residents’ fear of theft around leisure activity places (presumably because theft is less threatening) (*F*1), whereas prior victimization including theft in a bus or subway station and burglary in the community or an entertainment facility would increase the level of fear *F*1. These results show that people who have experience with severe crime have a higher threshold regarding the fear of theft and are less fearful. However, those who have experienced theft feel more likely to be stolen from in leisure activity spaces, demonstrating an increased fear of theft. In conjunction with these findings, gender (being female), high income groups and married couples likewise have higher rates of fear of theft around leisure places. This may be because they belong to more sensitive or wealthy groups. For example, married couples and wealthy people have nicer/more expensive things that could be taken, such as fancy wireless headphones.

In relation to *F*2, fear of theft around mid- to low-end places is positively related to burglary victimization, rape or murder happened in the community as well as the density of entertainment facilities. This may be because residents associate these crimes and locations with people who are active in low- and medium-end locations. For instance, thieves tend to be active in low- and medium-end locations due to lower guardianship, and this association increases the fear of theft. However, *F*2 is negatively related to the density of restaurants, hotels, cinemas and banks. This may be because such facilities are located in more populous areas and attract crowds, making residents more comfortable with busy environments and less sensitive to theft. Vulnerability variables have no significant impact, which indicates that there is no significant difference in *F*2 among groups with different socioeconomic attributes.

As for *F*3, fear of theft around high mobility places, among the measured vulnerability variables, only age is negatively significant showing that older people have a lower level of *F*3 fear than younger people. Prior experience with victimization has no significant impact on *F*3. These results show that regardless of socio-economic attributes and prior victimization experience, most residents have quite high fear levels around high mobility locations. In terms of community security, only burglary significantly increased residents’ level of *F*3. Interestingly, if there are more restaurants, cinemas or stadiums, residents have lower rates of fearing theft in high mobility locations. Yet, looking at a similar metric, the density of entertainment facilities and arts and culture venues leads to a rise in the fear of theft around high mobility locations. Further study is required to adequately determine the reasons for these differences.

## 6. Conclusions

The purpose of this study was to analyze fear of theft around different geographic locations and explain their impact factors, including vulnerability variables, victimization experience, community security and built environment factors in Guangzhou, China. The specific conclusions are as follows: (1) the respondents demonstrated significant location-based differences in their fear of theft. Locations including a coach station, a railway station, a bus station, a subway station and a wholesale market had the highest associated levels of fear, whereas locations dedicated to leisure activities, especially those in high-end places, had a lower level of respondents’ fear of theft. (2) Vulnerability model, victimization model, community security and built environment can be applied to the analysis of fear of theft around different places, but interpretations of fear do vary widely from place to place.

This study studies the fear of theft of residents around different locations, further enriches the literature of fear of crime from the perspective of space and shows that we should pay attention to the importance role of location and the crime type when studying fear of crime. The findings can also shed light on public policy to decrease people’s fear of crime around different places. For example, people have a higher level of fear of crime around bus stations and subway stations, and local police can improve the police visibility to make people feel safer.

There are some limitations to this study that should be highlighted. First, 12 types of POI density were used to represent the built environment in this study, but the constitution of the built environment may vary in different neighborhoods [50]. Second, this study explains residents’ location-based fear of theft and their impact factors. However, the impact factors can be quite complicated such as the level of guardianship in every place, and these have not been considered in this study. Finally, this study takes Guangzhou, a big city in the south of China, as an example to analyze the fear of theft in different locations and its influencing factors, but whether the findings can be applied to other cities such as those in the north still needs further research.

## Figures and Tables

**Table 1:** Measurement of fear of theft and its rotated component matrix.

Variable	Place	Mean	SD	*F*1	*F*2	*F*3
*Y*_1_	Parks, greenways and other recreational areas	2.815	1.037	0.807	-	-
*Y*_2_	Sports venues and surrounding areas	2.604	0.901	0.766	-	-
*Y*_3_	Internet cafes	3.209	1.030	0.715	-	-
*Y*_4_	Bars	3.232	1.044	0.700	-	-
*Y*_5_	KTV, cinema	2.944	0.975	0.666	-	-
*Y*_6_	High consumption restaurant	2.543	0.856	0.643	0.374	-
*Y*_7_	Medium consumption restaurant	2.604	0.861	0.480	0.301	-
*Y*_8_	Low consumption restaurant	2.710	0.900	-	0.856	-
*Y*_9_	Urban village	3.572	1.057	-	0.849	-
*Y*_10_	Bank and its surroundings	3.156	1.032	-	0.747	-
*Y*_11_	Everyday shopping places	2.881	0.936	-	0.634	0.311
*Y*_12_	Commercial Street/Shopping Center	3.175	1.010	0.541	0.565	-
*Y*_13_	Food market	3.107	0.996	-	0.506	-
*Y*_14_	Bus station, subway station	3.477	1.035	-	-	0.840
*Y*_15_	Wholesale market	3.254	0.994	-	-	0.839
*Y*_16_	Coach station, railway station	3.940	1.091	-	0.426	0.654

Note. The “-” indicates that the corresponding loads are less than 0.300.

**Table 2:** Measurement and descriptive statistics of correlates variables.

Variable Classification	Variable Code	Variable Name	Minimum	Maximum	Mean	SD
Vulnerability variables	*X*_1_	Gender	1.000	2.000	1.467	0.499
	*X*_2_	Age	19	89	38.030	14.016
	*X*_3_	Household income	1.000	6.000	1.750	1.079
	*X*_4_	Marital status	1.000	2.000	1.314	0.464
	*X*_5_	Hukou	1.000	2.000	1.564	0.496
Victimization experience	*X*_6_	Violence in community	1.000	5.000	1.169	0.479
	*X*_7_	Theft in community	1.000	5.000	1.237	0.553
	*X*_8_	Burglary	1.000	5.000	1.176	0.506
	*X*_9_	Theft in bus or subway	1.000	5.000	1.253	0.553
	*X*_10_	Theft in public places	1.000	5.000	1.232	0.542
Community security	*X*_11_	Theft	1.000	2.000	1.407	0.491
	*X*_12_	Burglary	1.000	2.000	1.339	0.473
	*X*_13_	Violence	1.000	2.000	1.189	0.391
	*X*_14_	Fraud	1.000	2.000	1.199	0.399
	*X*_15_	Rape or murder	1.000	2.000	1.060	0.237
Built environment (The density of POI)	*X*_16_	Bar	0.000	40.314	1.484	6.393
	*X*_17_	Restaurants	0.000	788.340	65.260	112.853
	*X*_18_	Hotels	0.000	60.471	10.079	15.225
	*X*_19_	Night clubs	0.000	1.764	0.143	0.406
	*X*_20_	Cinema	0.000	11.936	0.301	1.636
	*X*_21_	Entertainment facility	0.000	49.298	5.076	9.046
	*X*_22_	Scenic spots	0.000	62.520	5.509	10.897
	*X*_23_	Stores	0.000	335.045	42.255	57.274
	*X*_24_	Arts and culture venues	0.000	47.033	5.729	10.437
	*X*_25_	Stadium	0.000	32.958	3.898	7.150
	*X*_26_	Banks	0.000	194.608	20.740	35.578
	*X*_27_	Security facilities	0.000	47.033	5.295	9.904

Note. POI density is measured in units per square kilometer.

**Table 3:** Regression models for fear of theft in different locations.

Classification	Variable Code	Variable Name	Model 1		Model 2		Model 3		Collinearity Statistics	
			B	SE	B	SE	B	SE	Tol	VIF
Constant	*X*_0_	Constant	−0.143	0.259	−0.637	0.260	−0.114	0.254	-	-
vulnerability variables	*X*_1_	Gender	0.193 ***	0.051	0.097	0.051	0.067	0.050	0.942	1.062
	*X*_2_	Age	−0.002	0.002	−0.001	0.002	−0.013 ***	0.002	0.646	1.548
	*X*_3_	Household income	0.072 **	0.024	0.015	0.024	0.041	0.023	0.903	1.107
	*X*_4_	Marital status	−0.132 *	0.064	−0.004	0.064	0.099	0.063	0.678	1.474
	*X*_5_	Hukou	−0.015	0.053	−0.061	0.054	0.030	0.052	0.855	1.170
victimization experience	*X*_6_	Violence in community	−0.223 **	0.071	0.035	0.071	−0.003	0.070	0.518	1.932
	*X*_7_	Theft in community	0.039	0.069	−0.059	0.069	0.005	0.068	0.414	2.414
	*X*_8_	Burglary	−0.151 *	0.072	0.232 ***	0.073	0.008	0.071	0.448	2.233
	*X*_9_	Theft in bus or subway	0.134 *	0.067	−0.050	0.068	0.022	0.066	0.433	2.312
	*X*_10_	Theft in public places	0.015	0.073	0.090	0.074	−0.041	0.072	0.381	2.624
community security	*X*_11_	Theft	0.041	0.062	0.118	0.063	0.060	0.061	0.641	1.561
	*X*_12_	Burglary	0.244 ***	0.065	−0.114	0.065	0.185 **	0.064	0.634	1.578
	*X*_13_	Violence	−0.001	0.074	−0.027	0.074	0.027	0.073	0.718	1.392
	*X*_14_	Fraud	0.113	0.075	0.048	0.075	0.124	0.073	0.674	1.484
	*X*_15_	Rape or murder	−0.368 **	0.116	0.322 **	0.116	−0.160	0.114	0.795	1.257
built environment (The density of POI)	*X*_16_	Bar	0.000	0.000	0.000	0.000	0.000	0.000	0.206	4.863
	*X*_17_	Restaurants	0.005	0.005	−0.011 *	0.006	−0.019 ***	0.005	0.487	2.052
	*X*_18_	Hotels	0.004	0.003	−0.006 *	0.003	0.002	0.002	0.410	2.442
	*X*_19_	Night clubs	0.096	0.064	−0.011	0.064	0.058	0.063	0.888	1.126
	*X*_20_	Cinema	−0.005	0.016	−0.051 **	0.016	−0.031 *	0.016	0.845	1.183
	*X*_21_	Entertainment facility	0.009 **	0.004	0.015 ***	0.004	0.009 *	0.004	0.447	2.239
	*X*_22_	Scenic spots	0.002	0.003	0.003	0.003	−0.004	0.003	0.756	1.322
	*X*_23_	Stores	−0.001	0.001	0.000	0.001	0.000	0.001	0.356	2.809
	*X*_24_	Arts and culture venues	−0.003	0.005	0.004	0.005	0.016 ***	0.005	0.224	4.472
	*X*_25_	Stadium	−0.005	0.006	−0.009	0.006	−0.027 ***	0.006	0.372	2.691
	*X*_26_	Banks	−0.001	0.001	−0.003 **	0.001	−0.001	0.001	0.547	1.827
	*X*_27_	Security facilities	0.005	0.004	0.006	0.004	−0.005	0.004	0.429	2.330

Note: *** *p* < 0.001; ** *p* < 0.01; * *p* < 0.05; B is the regression coefficient; SE means standard errors. “-” indicates that the model does not contain the variable.

## Data Availability

Not applicable.

## References

[1] Sulemana I. (2015). The Effect of Fear of Crime and Crime Victimization on Subjective Well-Being in Africa. Soc. Indic. Res..

[2] Schafer J.A., Huebner B.M., Bynum T S. (2006). Fear of crime and criminal victimization: Gender-based contrasts. J. Crim. Justice.

[3] Mesch G.S. (2000). Perceptions of risk, lifestyle activities, and fear of crime. Deviant Behav..

[4] Nasar J.L., Fisher B. (1993). ‘Hot spots’ of fear and crime: A multi-method investigation. J. Environ. Psychol..

[5] Karakus O., Mcgarrell E.F., Basibuyuk O. (2010). Fear of crime among citizens of Turkey. J. Crim. Justice.

[6] Lee J., Cho S. (2018). The impact of crime rate, experience of crime, and fear of crime on residents’ participation in association: Studying 25 districts in the City of Seoul, South Korea. Crime Prev. Community Saf..

[7] Villarreal A., Yu W. (2017). Crime, fear, and mental health in Mexico. Criminology.

[8] Jing F., Liu L., Zhou S., Song G. (2020). Examining the relationship between hukou status, perceived neighborhood conditions, and fear of crime in Guangzhou, China. Sustainability.

[9] Zhang L., Messner S.F., Liu J., Zhuo Y.A. (2009). Guanxi and fear of crime in contemporary urban China. Br. J. Criminol..

[10] Cohen L.E., Felson M. (1979). Social Change and Crime Rate Trends: A Routine Activity Approach. Am. Sociol. Rev..

[11] Weisburd D., Amram S. (2014). The law of concentrations of crime at place: The case of Tel Aviv-Jaffa. Police Pract. Res..

[12] Malleson N., Andresen M.A. (2016). Exploring the impact of ambient population measures on London crime hotspots. J. Crim. Justice.

[13] Loughran T.A., Paternoster R., Chalfin A., Wilson T. (2016). Can rational choice be considered a general theory of crime? evidence from individual-level panel data. Criminology.

[14] Yates A., Ceccato V. (2020). Individual and spatial dimensions of women’s fear of crime: A Scandinavian study case. Int. J. Comp. Appl. Crim. Justice.

[15] Lee M.S., Ulmer J.T. (2000). Fear of crime among Korean Americans in Chicago communities. Criminology.

[16] Keane C. (1992). Fear of crime in Canada: An examination of concrete and formless fear of victimization. Can. J. Criminol..

[17] Chainey S., Tompson L., Uhlig S. (2008). The Utility of Hotspot Mapping for Predicting Spatial Patterns of Crime. Secur. J..

[18] Nakaya T., Yano K. (2010). Visualising Crime Clusters in a Space-time Cube: An Exploratory Data-analysis Approach Using Space-time Kernel Density Estimation and Scan Statistics. Trans. GIS.

[19] Cook C., Fox K. (2011). Fear of Property Crime: Examining the Effects of Victimization, Vicarious Victimization, and Perceived Risk. Violence Vict..

[20] Lin L., Guangwen S., Luzi X., Suhong Z., Guangqin S., Dongping L. (2018). Characteristics and Effect Factors of Victims’ Crime Reporting Behavior of Different Victimization. Sci. Geogr. Sin..

[21] Conklin J.E. (2014). Dimensions of Community Response to the Crime Problem. Soc. Probl. Probl..

[22] Garofalo J. (1981). The Fear of Crime: Causes and Consequences. J. Crim. Law Criminol..

[23] Ferraro K.F., Grange R.L. (1987). The Measurement of Fear of Crime. Sociol. Inq..

[24] Maslow A.H., Hirsh E., Stein M., Honigmann I. (1945). A Clinically Derived Test for Measuring Psychological Security-Insecurity. J. Gen. Psychol..

[25] Furstenberg F.F. (1971). Public Reaction to Crime in the Streets. Am. Sch..

[26] Lebowitz B.D. (1975). Age and fearfulness: Personal and situational factors. J. Gerontol..

[27] Baker M.H., Nienstedt B.C., Everett R.S., McCleary R. (1983). The Impact of a Crime Wave: Perceptions, Fear, and Confidence in the Police. Law Soc. Rev..

[28] Thomas C.W., Hyman J M. (1977). Perceptions of crime, fear of victimization, and public perceptions of police performance. J. Police Sci. Adm..

[29] Gray E., Jackson J., Farrall S. (2011). Feelings and Functions in the Fear of Crime: Applying a New Approach to Victimisation Insecurity. Br. J. Criminol..

[30] Gabriel U., Greve W. (2003). The psychology of fear of crime: Conceptual and methodological perspectives. Br. J. Criminol..

[31] Hale C. (1996). Fear of Crime: A Review of the Literature. Int. Rev. Vict..

[32] Oh J.H., Kim S. (2009). Aging, neighborhood attachment, and fear of crime: Testing reciprocal effects. J. Community Psychol..

[33] Abbott J., Mcgrath S.A. (2017). The Effect of Victimization Severity on Perceived Risk of Victimization: Analyses Using an International Sample. Vict. Offenders.

[34] Henson B., Reyns B.W., Fisher B.S. (2013). Fear of crime online? Examining the effect of risk, previous victimization, and exposure on fear of online interpersonal victimization. J. Contemp. Crim. Justice.

[35] Chadee D., Ying N.N., Chadee M., Heath L. (2016). Fear of crime: The influence of general fear, risk, and time perspective. J. Interpers. Violence.

[36] Tseloni A., Zarafonitou C. (2008). Fear of crime and victimization: A multivariate multilevel analysis of competing measurements. Eur. J. Criminol..

[37] Özascilar M., Ziyalar N. (2017). Unraveling the determinants of fear of crime among men and women in istanbul: Examining the impact of perceived risk and fear of sexual assault. Int. J. Offender Ther. Comp. Criminol..

[38] Werner G., Bernhard L., Cathleen K. (2017). Fear of crime in old age: A sample case of resilience?. J. Gerontol..

[39] Vauclair C.M., Bratanova B. (2017). Income inequality and fear of crime across the European region. Eur. J. Criminol..

[40] Liu J., Messner S.F., Zhang L., Zhuo Y. (2009). Socio-Demographic Correlates of Fear of Crime and the Social Context of Contemporary Urban China. Am. J. Community Psychol..

[41] Mao Y., Yin L., Zeng M., Ding J., Song Y. (2021). Review of Empirical Studies on Relationship between Street Environment and Crime. J. Plan. Lit..

[42] Jacobs J. (1961). The Death and Life of Great American Cities.

[43] Jeffery C.R. (1971). Crime Prevention Through Environmental Design.

[44] Wilson J.Q., Kelling G L. (1982). Broken windows: The police and neighborhood safety. Atl. Mon..

[45] Sampson R.J., Raudenbush S W. (2004). Seeing disorder: Neighborhood stigma and the social construction of “Broken Windows”. Soc. Psychol. Q..

[46] Wikström P.H., Dolmén L. (2022). Urbanisation, neighbourhood social integration, informal social control, minor social disorder, Mencken F C, Bader C D, Day L E. Fear of Crime on Community Engagement: Nonadditive and Nonlinear Effects by Gender. Sociol. Q..

[47] Song G., Liu L., Bernasco W., Zhou S., Xiao L., Long D. (2018). Theft from the person in urban China: Assessing the diurnal effects of opportunity and social ecology. Habitat Int..

[48] Singer A.J., Chouhy C., Lehmann P.S., Walzak J.N., Gertz M., Biglin S. (2018). Victimization, Fear of Crime, and Trust in Criminal Justice Institutions: A Cross-National Analysis. Crime Delinq..

[49] Song G., Liu L., He S., Cai L., Xu C. (2020). Safety perceptions among African migrants in Guangzhou and Foshan, China. Cities.

[50] Yang W., Cao X. (2018). Examining the effects of the neighborhood built environment on CO_2_ emissions from different residential trip purposes: A case study in Guangzhou, China. Cities.

